# Robot-assisted minimally invasive thoraco-laparoscopic esophagectomy versus minimally invasive esophagectomy for resectable esophageal adenocarcinoma, a randomized controlled trial (ROBOT-2 trial)

**DOI:** 10.1186/s12885-021-08780-x

**Published:** 2021-09-26

**Authors:** E. Tagkalos, P. C. van der Sluis, F. Berlth, A. Poplawski, E. Hadzijusufovic, H. Lang, M. I. van Berge Henegouwen, S. S. Gisbertz, B. P. Müller-Stich, J. P. Ruurda, M. Schiesser, P. M. Schneider, R. van Hillegersberg, P. P. Grimminger

**Affiliations:** 1 Department of General-, Visceral- and Transplant Surgery, University Medical Center of the Johannes Gutenberg University, Langenbeckstrasse 1, D-55131 Mainz, Germany; 2 Institute for Medical Biometry, Epidemiology and Informatics (IMBEI), University Medical Center of the Johannes Gutenberg University, Mainz, Germany; 3 Department of Surgery, Cancer Center Amsterdam, Amsterdam UMC, University of Amsterdam, Amsterdam, The Netherlands; 4 Department of General, Visceral, and Trauma Surgery, University of Heidelberg, Heidelberg, Germany; 5 Department of Surgery, University Medical Center Utrecht, Utrecht, The Netherlands; 6 Chirurgisches Zentrum Zürich, Klinik Hirslanden, Zürich, Switzerland

**Keywords:** MIE, RAMIE, Ivor-Lewis, Esophageal adenocarcinoma, Lymphadenectomy

## Abstract

**Background:**

For patients with esophageal adenocarcinoma or cancer of the gastroesophageal junction, radical esophagectomy with 2-field lymphadenectomy is the cornerstone of the multimodality treatment with curative intent. Both conventional minimally invasive esophagectomy (MIE) and robot assisted minimally invasive esophagectomy (RAMIE) were shown to be superior compared to open transthoracic esophagectomy considering postoperative complications. However, no randomized comparison exists between MIE and RAMIE in the Western World for patients with esophageal adenocarcinoma.

**Methods:**

This is an investigator-initiated and investigator-driven multicenter randomized controlled parallel-group superiority trial. All adult patients (age ≥ 18 and ≤ 90 years) with histologically proven, surgically resectable (cT1-4a, N0–3, M0) esophageal adenocarcinoma of the intrathoracic esophagus or adenocarcinoma of the gastroesophageal junction and with European Clinical Oncology Group performance status 0, 1 or 2 will be assessed for eligibility and included after obtaining informed consent. Patients (*n* = 218) with resectable esophageal adenocarcinoma of the intrathoracic esophagus or adenocarcinoma of the gastroesophageal junction are randomized to either RAMIE (*n* = 109) or MIE (*n* = 109). The primary outcome of this study is the total number of resected abdominal and mediastinal lymph nodes specified per lymph node station.

**Conclusion:**

This is the first randomized controlled trial designed to compare RAMIE to MIE as surgical treatment for resectable esophageal adenocarcinoma or adenocarcinoma of the gastroesophageal junction in the Western World. The hypothesis of the proposed study is that RAMIE will result in a higher abdominal and mediastinal lymph node yield specified per station compared to conventional MIE.

Short-term results and the primary endpoint (total number of resected abdominal and mediastinal lymph nodes per lymph node station) will be analyzed and published after discharge of the last randomized patient within this trial.

**Trial registration:**

ClinicalTrials.gov Identifier: NCT04306458. Registered 13th March 2020, https://clinicaltrials.gov/ct2/show/NCT04306458; Date of first enrolment 18.01.2021; Target sample size 218; Recruitment status: Recruiting; Protocol version 2; Issue date 10.03.2020; Rev. 02.02.2021; Authors ET, PCvdS, PPG.

## Background

Esophageal cancer is the sixth leading cause of death from cancer worldwide with an estimated 400,000 new cases annually [1]. A transthoracic esophagectomy with abdominal and thoracic lymph node dissection is the cornerstone of the multimodality treatment with curative intent for patients with esophageal cancer or cancer of the gastroesophageal junction [2–5].

Until now, there are 4 randomized controlled trials which compared minimally invasive esophagectomy (MIE) to open transthoracic esophagectomy (OTE). In the TIME-trial, conventional thoraco-laparoscopic MIE was compared to OTE [6]. MIE resulted in a lower incidence of pulmonary infections and quality of life compared to OTE [6, 7]. In the MIRO trial, hybrid esophagectomy (HE) was compared to OTE. HE resulted in a lower incidence of major complications [8]. In the MIOMIE-trial, HE was compered to OE [9]. Here MIE and OE were equal as far as morbidity, mortality and oncological long-term results were compared. In the ROBOT trial, robot-assisted minimally invasive esophagectomy (RAMIE) was compared to OTE and resulted in a lower percentage of overall surgery-related and cardiopulmonary complications with lower postoperative pain resulting in better short-term postoperative functional recovery and better quality of life [10]. Aforementioned randomized controlled trials all show a benefit of minimally invasive esophagectomy over OTE considering postoperative complications and quality of life.

RAMIE facilitates complex minimally invasive procedures with an enlarged 3-dimensional view. The articulated instruments allow dissection with 7 degrees of freedom. RAMIE facilitates precise dissection in the narrow working space in the posterior mediastinum and it is technically feasible and safe in terms of oncological outcomes [11, 12].

Until now there is one meta-analysis which compared RAMIE to MIE [13]. This meta-analysis included 8 case control studies and no randomized controlled trials. In this meta-analysis included 3 Western studies [14–16]. It was concluded that RAMIE and MIE display similar effects and safety in the treatment of esophageal cancer. However, RAMIE could reduce the risk of laryngeal recurrent nerve damage due to improved vision and flexibility during the paratracheal lymph node dissection [13]. In a retrospective cohort analysis performed by our group 50 RAMIE procedures were compared to 50 MIE procedures [17]. Compared to MIE, RAMIE showed improved lymphadenectomy compared to conventional MIE (27 versus 23 lymph nodes, *p* = 0.043) [17]. It was decided that the primary endpoint for the randomized controlled trial (ROBOT-2 trial; RAMIE versus MIE for esophageal adenocarcinoma) is the extent of lymph node dissection. Until now, there are no prospective randomized controlled trials comparing RAMIE to MIE for patients with resectable intrathoracic esophageal adenocarcinoma in Western World patients. The hypothesis of this study is that RAMIE will lead to an improved lymph node dissection compared to MIE. We present the protocol of the first randomized controlled trial comparing these two surgical approaches in the Western World.

## Methods / design

### Aim of the study

This is a randomized controlled parallel-group, superiority trial comparing RAMIE to MIE with intrathoracic anastomosis (Ivor-Lewis) in patients with resectable intrathoracic esophageal adenocarcinoma or adenocarcinoma of the gastroesophageal junction in the Western World. The aim of this study is to compare abdominal and mediastinal lymphadenectomy per lymph node station as stated by the TIGER trial [18].

### Study design

Patients with resectable esophageal adenocarcinoma or adenocarcinoma of the gastroesophageal junction are randomized at the outpatient department to either (a) robot-assisted minimally invasive esophagectomy (RAMIE) or (b) conventional minimally invasive esophagectomy (MIE). This is a multicenter investigator-initiated and investigator-driven randomized controlled parallel-group, superiority trial comparing RAMIE to MIE. This study is conducted in accordance with the principles of the Declaration of Helsinki [19] and Good Clinical Practice Guidelines [20]. The independent ethics committee of the University Medical Center of the Johannes Gutenberg University, Mainz, Germany has approved the study (Nr: 2019–14,049). The World Health Organisation Trial Registration Data Set is shown in Table 1. Written informed consent will be obtained from all participating patients. Any modifications to the protocol which may impact on the conduct of the study, or may affect patient safety, including changes of study objectives, study design, patient population, sample sizes, study procedures, or significant administrative aspects will require a formal amendment to the protocol. Such amendment must be approved by the ethics committee prior to implementation. All centers participating in the ROBOT-2 trial have extensive experience in both RAMIE and MIE and have an experience with at least 50 MIE and 50 RAMIE procedures performed per center (high volume centers).

Clinical trial monitoring will be conducted by an independent data monitor from the Johannes Gutenberg University, Mainz in Germany. The committee is independent from the sponsor and competing interests. A Data safety monitoring board (DSMB) will evaluate safety for patients included within this trial. All outcomes will be evaluated by a (blinded) external independent data committee board for the Upper GI International Robotic association (UGIRA; www.urgira.org).

### Study population

**Fig. 1:**
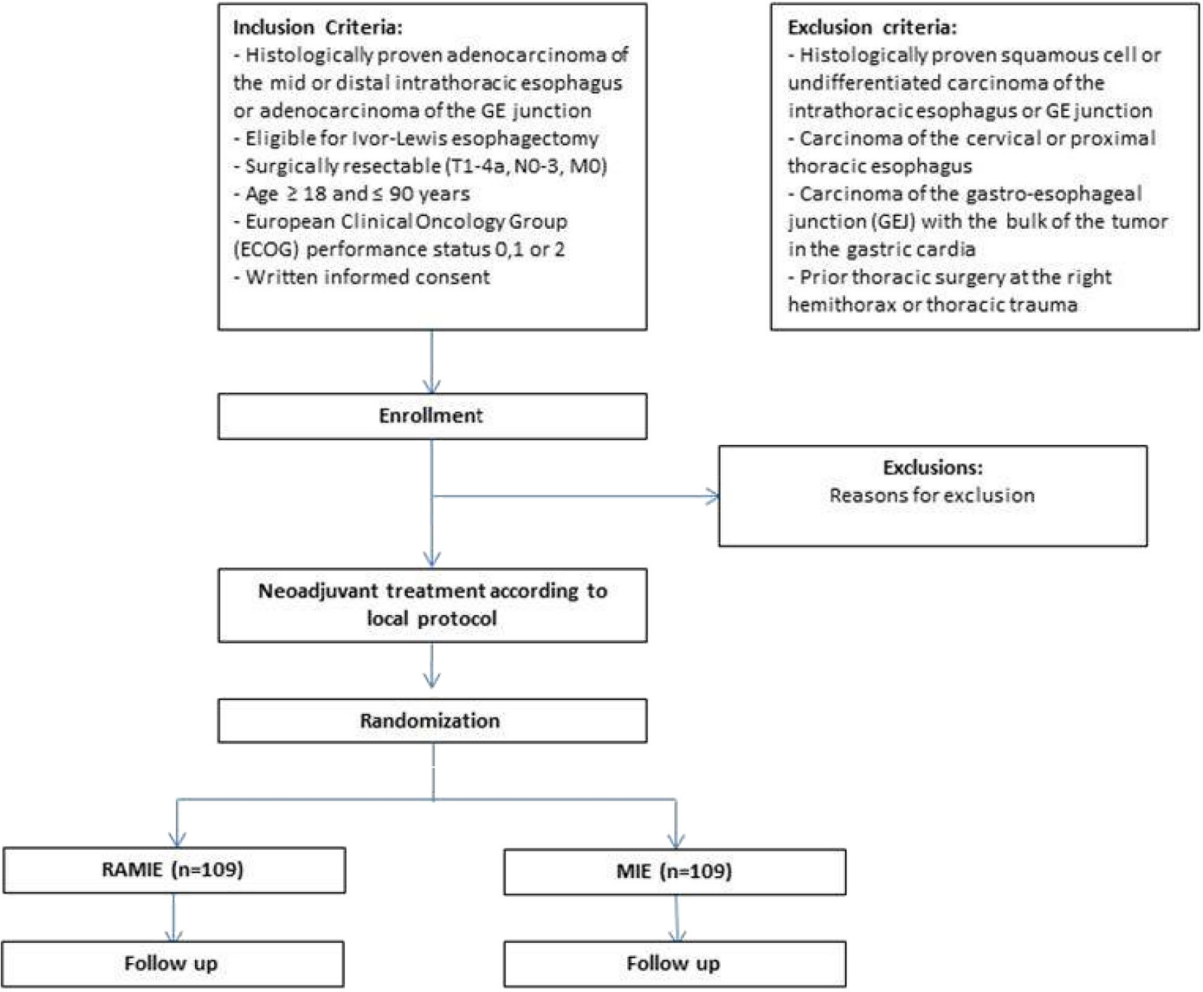
Flowchart for the ROBOT-2 trial

All adult patients (age ≥ 18 and ≤ 90 years) with histologically proven, surgically resectable (cT1-4a, N0–3, M0) adenocarcinoma of the mid or distal intrathoracic esophagus or adenocarcinoma of the GE junction will be assessed for eligibility. Patients should have a performance status 0, 1 or 2 according to the European Clinical Oncology Group (ECOG) (Table 2). The trial flowchart was shown in Fig. 1.

**Table 1:** World Health Organization Trial Registration Data Set

Data category	Information
Primary registry and trial identifying number	ClinicalTrials.gov NCT04306458
Date of registration in primary registry	13th March, 2020
Secondary identifying numbers	ROBOT2 trial, 2019–14,049
Source of material support	Intuitive Surgical Inc.
Primary sponsor	Intuitive Surgical Inc.
Contact for public queries	ET, PPG [email address]
Contact for scientific queries	ET, PPG University Medical Center Mainz, Mainz, Germany
Public title	RAMIE Versus MIE for Resectable Esophageal Cancer, a Randomized Controlled Trial (ROBOT-2 Trial).
Scientific title	Randomized controlled parallel-group, superiority trial comparing RAMIE to MIE with intrathoracic anastomosis (Ivor-Lewis) in patients with resectable intrathoracic esophageal adenocarcinoma or adenocarcinoma of the gastroesophageal junction in the Western World.
Countries of recruitment	Germany, The Netherlands, Switzerland
Health condition(s) or problem(s) studied	Experimental: Robotic assisted minimally invasive esophagectomy (RAMIE) for esophageal cancer
Intervention(s)	Active comparator: Minimally invasive esophagectomy (MIE) for esophageal cancer
Key inclusion and exclusion criteria	Ages eligible for study: 18–90 years of age Sexes eligible for study: both Accepts healthy volunteers: no Inclusion criteria: Histologically proven adenocarcinoma of the intrathoracic esophagus and gastroesophageal junction (including Siewert types I and II) Surgically resectable (T1-4a, N0–3, M0) European Clinical Oncology Group (ECOG) performance status 0,1 or 2 Written informed consent Exclusion criteria: Esophageal squamous cell carcinoma Carcinoma of the cervical esophagus Carcinoma of the esophageal junction (GEJ) with the main part of the tumor in the gastric cardia (Siewert type III) Prior thoracic surgery at the right hemithorax or thoracic trauma
Study type	Interventional Allocation: randomized; Intervention model: parallel assignment; Masking: single (outcomes assessor); Masking description: anonymous cases Primary purpose: treatment
Date of first enrolment	18th January 2021
Target sample size	218
Recruitment status	Recruiting
Primary outcome(s)	Total number of dissected lymph nodes in the resection specimen according to the TIGER classification
Key secondary outcomes	1.Postoperative complications [Time Frame: Operation date till date of discharge until 52 weeks postoperatively] 2.Length of intensive care unit (ICU) and hospital stay [Time Frame: Operation date till date of discharge until 52 weeks postoperatively] 3.In hospital mortality (IHM) [Time Frame: Hospital admission period up to 90 days postoperatively] 30, 60 and 90 day mortality 4.Pathology results [Time Frame: Up to 2 weeks postoperatively] Radical resection (R0 and R1) 5.Survival [Time Frame: 5 years postoperatively] Overall and disease free survival (2,3 and 5 year) 6.Operation statistics [Time Frame: day of operation] Operating time (thoracic, abdominal and total), blood loss, intraoperative complications 7.Postoperative pain [Time Frame: Before operation (baseline), daily during admission in the first 14 days, postoperatively: 6 weeks, 6 months and yearly post-operatively up to 5 years] Postoperative pain scores on a visual analogue scale (VAS) 8.Cost analysis [Time Frame: date of operation until 1 year postoperatively] Cost analysis 9.Surgeons fatigue [Time Frame: Day of operation] Surgeons fatigue directly after Operation assessed by Psychomotor Vigilance tests (PVT) before and after esophagectomy 10.Quality of life after esophagectomy [Time Frame: Before operation (baseline), at discharge, postoperatively: 6 weeks, 6 months and yearly up to 5 years post-operatively] Quality of life assessed by questionnaire European Organisation for Research and Treatment of Cancer (EORTC) QLQ-C30 11.Postoperative Recovery [Time Frame: 14 days postoperatively] Dutch discharge criteria (removal of thoracic tubes, no requirement of intravenous fluid resuscitation, tolerance for solid oral intake, the ability to mobilize independently and adequate pain control with oral analgesics) 12.Quality of life after esophagectomy [Time Frame: Before operation (baseline), at discharge, postoperatively: 6 weeks, 6 months and yearly up to 5 years post-operatively] Quality of life assessed by questionnaire European Organisation for Research and Treatment of Cancer (EORTC OES18) 13.Quality of life after esophagectomy [Time Frame: Before operation (baseline), at discharge, postoperatively: 6 weeks, 6 months and yearly up to 5 years post-operatively] Quality of life assessed by questionnaire Short Form (SF)-36 14.Quality of life after esophagectomy [Time Frame: Before operation (baseline), at discharge, postoperatively: 6 weeks, 6 months and yearly up to 5 years post-operatively] Quality of life assessed by questionnaire EuroQol (EQ)-5D

**Table 2:** Patients’ in- and exclusion criteria

**Inclusion criteria**	
- Histologically proven adenocarcinoma of the mid or distal intrathoracic esophagus or adenocarcinoma of the GE junction	
- Eligible for Ivor-Lewis esophagectomy	
- Surgically resectable (T1-4a, N0–3, M0)	
- Age ≥ 18 and ≤ 90 years	
- European Clinical Oncology Group (ECOG) performance status 0,1 or 2	
- Written informed consent	
**Exclusion criteria**	
- Carcinoma of the cervical or proximal thoracic esophagus	
- Carcinoma of the gastro-esophageal junction (GEJ) with the bulk of the tumor in the gastric cardia	
- Prior thoracic surgery at the right hemithorax or thoracic trauma

**Table 3:** Follow up assessment

Assessment	30 days after OP	6 weeks after OP	90 days after OP	6 months after OP	12 months after OP	18 months after OP	24 months after OP	36 months after OP	48 months after OP	60 months after OP
QoL Questionnaires		x		x	x		x	x	x	x
Clinical examination	x		x	x	x	x	x	x	x	x
VAS for pain		x		x	x		x	x	x	x

### Study protocol

Patients are informed about the trial by one of the surgeons at the outpatient department. After receiving the patient information, all patients have 1-week time to consider their consent. After obtaining informed consent, block randomization (according to prior therapy and according to the nodal status), with concealment of allocation, is done centrally by an online randomization program for each and every center. All patients who give consent for participation and who fulfil the inclusion criteria will be randomized. Randomization will be requested by the staff member responsible for recruitment and clinical interviews. Patients will be blinded for the allocated treatment (RAMIE or MIE). The surgeon and coordinating researcher will not be blinded because this is difficult in daily practice. However, UGIRA which provides surgical quality control by analyzing photographs of the dissected lymph node stations and the data safety monitoring board (DSMB) will be blinded to the allocated intervention. All resection specimens with separate lymphadenectomy stations will be preserved and stored (biobank) for translational research (a consent from must be filled in separately for each participant). The access to the coding list and thus to a potential decryption lies solely with the employees / study directors involved in the study. A consultation by the data protection officer of the University Medical Center Mainz took place before the study. All study-related information will be stored securely at the study site. All participant information will be stored in locked file cabinets in areas with limited access. All laboratory specimens, reports, data collection, process, and administrative forms will be identified by a coded identification number (ID) only to maintain participant confidentiality. All records that contain names or other personal identifiers, such as locator forms and informed consent forms, will be stored separately from study records identified by code number. All local databases will be secured with password-protected access systems. Forms, lists, logbooks, appointment books, and any other listings that link participant ID numbers to other identifying information will be stored in a separate, locked file in an area with limited access. All data between the centers involved will be pseudonymized. Participants’ study information will not be released outside of the study without the written permission of the participant, except from the data that are required from the national cancer register. The data will be archived for 10 years after the end of the study.

In case of absolute necessity, a code break can occur (unbinding). The randomization program contains such a mechanism if such circumstances are raised. All such cases (code breaks) will be reported (with the reason for the break code) at the prime investigator of the University Medical Center Mainz, which is responsible for the unblinding function.

The study started on January 18th 2021. Inclusion will take approximately 2 years. Follow up for each patient will be 5 years. Total duration of the study will be 7 years.

### Neoadjuvant and perioperative treatment

Patients with locally advanced disease (cT1 cN+ and cT2–4 Nx cM0) will be treated with neoadjuvant chemoradiotherapy (according to the CROSS protocol; carboplatin, paclitaxel and concurrent 41.4 Gy radiotherapy) or perioperative chemotherapy (according to the FLOT protocol; 5-FU, Leucovorin, Oxaliplatin, Docetaxel). Neoadjuvant chemoradiotherapy or perioperative chemotherapy will be administered according to local national policy[21–23] Individual changes in the theraputhic regime are also accepted after approval of the oncological board.

### Surgery

All procedures (RAMIE or MIE) will be carried out by the same experienced surgeons in the University Medical Center of the Johannes Gutenberg University (Mainz, Germany), Amsterdam UMC of the University of Amsterdam Cancer Center (Amsterdam, the Netherlands), the Department of General, Visceral, and Trauma Surgery of the University of Heidelberg (Heidelberg, Germany), the Department of Surgery of the University Medical Center Utrecht (Utrecht, The Neatherland) and the Surgical Center Zurich, (Zürich, Switzerland). Local pre-, peri- and postoperative protocols will be followed. Both surgical procedures (MIE and RAMIE) should be standard of care in the participating centers with the required experience by the performing surgeon. The required minimum of performed operations is based on published learning curve data and determined by the group as follows: A minimum of 50 performed RAMIE cases per surgeon to include patients in the RAMIE arm and a minimum of 50 performed MIE cases per surgeon to include in the MIE arm.

All patients will receive an epidural or paravertebral catheter to provide adequate postoperative analgesia. Patients will be intubated with a left-sided double-lumen tube to enable selective desufflation of the right lung during the thoracic phase in both procedures. Antibiotic prophylaxis will be administered 30 min prior to incision according to local protocol. A chest tube or Jackson Pratt drain will be positioned in the right hemithorax at the end of the procedure. Extubating will take place in the operating theater directly postoperatively and hereafter all patients will be admitted to the intensive care unit (ICU), medium care unit (MCU) for hemodynamic and respiratory monitoring. Hemodynamical and respiratory stable patients will be discharged towards the surgical ward on the first day postoperatively.

There are no restricted interventions if needed. Moreover participants should continue to take medication for other conditions as normal.

### Surgical procedure: RAMIE

The RAMIE technique using the 4 arm daVinci Xi system was described previously (daVinci Xi system, Intuitive Surgical Inc., Sunnyvale, CA, USA) [24]. For the abdominal phase, the patient is placed in supine position. Robotic trocars are positioned according to local protocol. The lesser omentum is opened and transected closely to the liver, until the left crus of the diaphragm is reached. The greater gastric curvature is dissected. The left gastric artery and vein are ligated and transected at their origin. An abdominal lymphadenectomy is performed (see paragraph: primary endpoint). The gastric conduit is created at the level of the angular incisure using an endostapler.

For the thoracic phase, the patient is positioned in the left lateral decubitus position, tilted 45° towards the prone position (semi-prone). Robotic trocars are positioned according to local protocol. The robotic system is brought into the field at the dorsocranial side of the patient. After incision and installation of the operation robot and selective desufflation of the right lung, the pulmonary ligament is divided. Hereafter, the parietal pleura is dissected at the anterior side of the esophagus from the diaphragm up to the azygos arch. The azygos vein is ligated and divided. Dissection of the parietal pleura is continued above the azygos arch to establish dissection of the right paratracheal lymph nodes. At the posterior side of the esophagus, the parietal pleura is dissected cranially to caudally along the azygos vein, including the thoracic duct. The thoracic duct is clipped. The esophagus is resected en bloc with the surrounding mediastinal lymph nodes (see paragraph: primary endpoint).

The gastric conduit is pulled up and the specimen is removed through a small incision (mini-thoracotomy) at the location of the trocar in the 6th intercostal space. Continuity is created using a stapled esophago-gastrostomy.

### Surgical procedure: MIE

For the abdominal phase, the patient is placed in supine position. Abdominal trocars are positioned according to local protocol. The lesser omentum is opened and transected closely to the liver, until the left crus of the diaphragm is reached. The greater gastric curvature is dissected. An abdominal lymphadenectomy is performed (see paragraph: primary endpoint). The left gastric artery and vein are ligated and transected at their origin. The gastric conduit is created at the level of the angular incisure using an endostapler.

For the thoracic phase, the patient is positioned in the left lateral decubitus position, tilted 45° towards the prone position (semi-prone) or prone position. Thoracic trocars are positioned according to local protocol. If the patient is in semi-prone, selective desufflation of the right lung is performed, for patients in prone the right lung remains ventilated. The pulmonary ligament is divided. Hereafter, the parietal pleura is dissected at the anterior side of the esophagus from the diaphragm up to the azygos arch. The azygos vein is ligated [25]. Dissection of the parietal pleura is continued above the azygos arch to establish dissection of the right paratracheal lymph nodes. At the posterior side of the esophagus, the parietal pleura is dissected cranially to caudally along the azygos vein, including the thoracic duct. The thoracic duct is clipped. The esophagus is resected en bloc with the surrounding mediastinal lymph nodes (see paragraph: primary endpoint). The gastric conduit is pulled up and the resection specimen is removed through a small incision (mini-thoracotomy) Continuity is created using a stapled esophago-gastrostomy.

### Primary endpoint

The primary outcome of this study is the total number of dissected abdominal and mediastinal lymph nodes specified per station as stated by the TIGER-trial. The resected specimen will be marked by the surgical team for the location of the lymph node stations.

An abdominal lymphadenectomy is performed (including paracardial lymph nodes (station 14 R and L), lymph nodes along the left gastric artery (station 15), the celiac trunk (station 16), the splenic artery (station 17), the common hepatic artery (station 18) and hepatoduodenal ligament (station 19) [18].

Mediastinal lymphadenectomy will include: lower paratracheal right (station 7), subcarinal (station 9), paraesophageal (station 10–12) and pulmonary ligament (station 13) lymph nodes. Upper paratracheal (station 6) and the aortopulmonary window (station 8) lymphadenectomy will only be performed when lymph node metastases are suspected at these stations [18]. All lymph node stations will be sent as separate items for pathological examination. These separate lymph node stations can be obtained intraoperatively or with back table dissection postoperatively by the operating surgeon. After lymphadenectomy, photographs of each separate dissected lymph node station will be taken for surgical quality control.

These photos will be analyzed centrally by UGIRA. Evaluation of the resection specimen will be performed by an experienced GI-pathologist using a standard protocol (the TIGER-trial abdominal and mediastinal stations) [18] used in all centers. Stage grouping will take place according to the Union Internationale Contre le Cancer (UICC) protocol using the TNM-8 classification [25].

### Secondary outcomes include

- Overall postoperative complications according to the modified Clavien Dindo classification (MCDC grade I-V)) [26] and definitions stated by the Esophagectomy Complications Consensus Group (ECCG) [27]. Postoperative complications include: anastomotic leakage, mediastinitis, gastric conduit necrosis, chylothorax and recurrent laryngeal nerve injury, delayed gastric emptying, pulmonary complications (pneumonia, pneumothorax, pulmonary embolus, acute respiratory distress syndrome (ARDS)), cardiac complications (atrial fibrillation, cardiac asthma, myocardial infarction) and postoperative bleeding. The incidence of incisional hernias and diaphragmatic hernias will be recorded.
- Complications as stated by the comprehensive complication index (CCI) [28] and textbook outcome [29].
- Length of ICU-MCU stay (days), length of hospital stay (days)
- In hospital mortality (IHM) and mortality within 30, 60 and 90 days postoperatively will be reported. For all patients, the cause of death will be noted.
- Pathology results: The pathology report contains the following parameters: site and size of tumor, type and gradation, extension in the esophageal wall, margins of the resection, extent of resection (R0, R1 or R2), lymph node status with the total number of lymph nodes and the number of positive lymph nodes per lymph node station (TNM 8 and TIGER) [25]. A standard protocol will be used in all centers.
- Overall and disease-free survival (2, 3 and 5 years). Overall survival (OS) was calculated from the date of surgery to the date of death or last follow-up. Disease free survival was calculated from the date of surgery to recurrence or death related to disease and/or treatment or last date of follow-up.
- Operation time is defined as time from incision until closure (minutes) for both the thoracic and the abdominal phase of the procedure. Unexpected events and complications occurring during the operation will be recorded (e.g. hemorrhage requiring transfusion, perforation of other organs) as well as blood loss during operation (ml, per phase). In case of conversion to thoracotomy or laparotomy the reason for conversion has to be explained (absolute numbers/percentage).
- Visual Analogue Scale (VAS) for pain will be noted at following times: pre-operatively and the first 14 days after surgery and at fixed intervals during follow up (6 weeks, 6 months and yearly post-operatively up to 5 years).
- Quality of life questionnaires (QoL) will be required at following times: SF-36, EORTC QLQ-C30 (German), EORTC OES18 (German) and EQ-5D (Appendix 1 & 2) pre-operative < 5 days and 6 weeks, 3 months, 6 months and yearly up to 5 years post-operatively [30].
- Postoperative functional recovery within 14 days and within the period of hospitalization. Postoperative recovery is defined as: removal of thoracic tubes, no requirement of intravenous fluid resuscitation, tolerance for solid oral intake, the ability to mobilize independently and adequate pain control with oral analgesics. All items will be assessed daily.
- Cost analysis: The approach for the cost-analysis is comparing actual direct medical costs incurred with both strategies up until 5 years after the operation. Costs estimates will be based on the recorded volumes and unit costs associated with both procedures. This includes the costs of operation rooms, hospital and ICU stay, costs associated with complications and re-operations.
- Surgeons fatigue assessed by Psychomotor Vigilance tests (PVT) before and after esophagectomy.
- Conversion

To further improve monitoring adherence the posoperative patient’s visits are planned 3 months, 6 months, 12 months, 18 months, 24 months, 36, month, 48 months and 60 months after the operation (Table 3). The patients will be contacted prior to the planned visits and from the study assistents, as well as periodically in order to minimise loss of data and facilitate a thorough follow up. Loss-to-follow-up is expected to be at most 5%. The oncological follow-up of the patients will be conducted as for every patient that is undergoing surgury for esophageal cancer cancer according to the national guidelines.

### Data collection methods and management

All data will be collected from trained physicians and will be controlled from the PI or SI in Mainz and the lead investigators in the other centers (Amsterdam, Utrecht, Heidelberg and Zurich). The documentation of the VAS for will be conducted daily (at the same time every day in written form) and in specified intervals from the trained physicians (after further training in the study requirements). Moreover the QoL questionnaires will be filled from the patients with the help of trained physicians (after further training). The data to be collected and the procedures to be conducted at each visit will be reviewed in detail. Each of the data collection forms and the nature of the required information will be discussed in detail on an item by item basis. After thorough control of the handwritten data from the lead investigators, the upload of the data in the electronic databases will be conducted through trained physicians and study assistants.

Data integrity will be enforced through a variety of mechanisms. The data entry screens are user friendly and the option to choose a value from a value list of valid codes is given. Modifications to data inserted in the databases will be documented and explained. Missing data or errors will be reported from the study assistants to the lead investigators in order to be clarified. The hardware in which the databases will be kept will be locked and access to this is restricted. The type of activity that an individual user may undertake is regulated by the privileges associated with his/her user identification code and password. Back-ups of the master data file will also be kept.

### Criteria for discontinuing or modifying allocated interventions for a member participant

At any time the participant can withdraw his/her consent to participate in the study (verbal or in writing). For a given trial participant, the assigned study intervention may need to be modified or discontinued by trial investigators for various reasons, including harms, lack of efficacy, or alternation on the patient’s condition. Regardless of any decision to modify or discontinue their assigned intervention, study participants should be retained in the trial whenever possible to enable follow-up data collection and prevent missing data.

### Adverse events, unintended events or trial conduct, auditing for trial conduct

The collecting, assessing, reporting, and managing solicited and spontaneously reported adverse events or other unintended effects of trial interventions or trial conduct would be collected in each center and would be reported to the University Medical Center Mainz.

Auditing for trial conduct, if any, would be conducted independent from the investigators and the sponsor upon the occurrence of such conduct.

### Sample size calculation

The sample size calculation is based on a prospective analysis of lymph node dissection in 50 RAMIE and 50 MIE patients in Mainz. Compared to MIE, RAMIE showed improved lymphadenectomy compared to conventional MIE (27 versus 23 lymph nodes, *p* = 0.043) [17]. The effect size was determined at 0.4 and it was calculated that 104 patients (208 in total) in each arm with resectable esophageal adenocarcinoma or adenocarcinoma of the gastroesophageal junction would be required based on a two-sided significance level (alpha) of 0.05 and a power of 0.80. An estimated compensation of 5% for drop out was included in the total number of patients, resulting in a total of 218 patients, 109 in each arm.

### Statistical analysis

All prospective data will be statistically analyzed by the use of the statistical software SPSS (Chicago, IL). All analyses will be performed according to the intention-to-treat (ITT) principle. Results will be presented as risk ratios with corresponding 95% confidence intervals (CI). To evaluate significance of differences between groups, the chi-squared test was used as appropriate for categorical variables and the student’s T-test and non-parametric Mann-Whitney U-test for continuous variables.

Differences over time in quality of life and pain scores between and within treatment groups will be assessed using linear mixed-effects models adjusted for the baseline value. Overall and progression-free survival curves were estimated with the Kaplan-Meier method and compared with the log-rank test. All reported *P*-values were two-sided. Significance level is set at 0.05.

The cost-effectiveness analysis will compare the mean costs and effects for both strategies and result in an incremental cost-effectiveness ratio. Uncertainty in the balance between costs and effects will be assessed with bootstrapping. A time horizon of 5 years will be applied, and costs and effects will be discounted according to international guidelines.

If the baseline characteristics differ after randomization, i.e. there is a lack of balance in the confounding factors; this will be corrected using the multivariate analysis or by using a net benefit regression approach.

### Interim-analysis

There will be one interim-analysis. The stopping rule used for efficacy (i.e. better outcome for minimally invasive for the primary endpoint) is the Peto-approach, meaning a *p*-value < 0.001. The trial will not be stopped for futility (i.e. no difference) and there is no formal stopping rule for harm.

After every 50 patients, individualized patient description charts including safety parameters will be presented to the UGIRA Data Safety Monitoring Board (DSMB). The DSMB will discuss these in a plenary, telephone or online conference with the study coordinator and principal investigator present. The trial research group will discuss in a plenary session together with the DSMB the potential harm per patient and determine whether a relationship can be drawn between the surgical procedure and the adverse events. Consensus will be reached and the Institutional ethical board will be informed.

## Discussion

This is the first randomized controlled trial designed to compare robot-assisted minimally invasive thoraco-laparoscopic esophagectomy (RAMIE) with conventional minimally invasive esophagectomy in the Western World as surgical treatment for resectable esophageal adenocarcinoma.

There is considerable evidence that both MIE and RAMIE are alternatives to the standard open esophagectomy for esophageal cancer. However, the question remains, whether the technical advantages of RAMIE contribute to better results compared to conventional MIE.

In Asian studies, where RAMIE was compared to MIE, a higher mean lymph node yield along the recurrent laryngeal nerve was observed in favor of RAMIE [31–34]. Furthermore, RAMIE showed a reduced rate of recurrent laryngeal nerve injury compared to MIE [13]. In a retrospective cohort analysis performed in Mainz 50 RAMIE procedures were compared to 50 MIE procedures [17]. Compared to MIE, RAMIE showed improved lymphadenectomy compared to conventional MIE (27 versus 23 lymph nodes, *p* = 0.043) [17]. The technical superiority of RAMIE over MIE due to the 3-dimensional 10-times enlarged image combined with the excellent dexterity might results in an improved lymph node dissection and possibly less recurrent laryngeal nerve injuries.

A Population-based Cohort Study in the Netherlands, including 2698 patients showed that a higher lymph node yield was significantly associated with improved overall survival, indicating a therapeutic value of extended lymphadenectomy during esophagectomy [35]. With possible better lymphadenectomy with RAMIE, overall and disease-free survival are important secondary endpoints in the ROBOT-2 trial even though probably this trial is underpowered to show a difference in survival.

Until now, the 3 most important trials showing superiority of MIE over OTE had postoperative (pulmonary) complications as primary endpoint [6, 8, 10]. However, within the ROBOT-2 trial, since both techniques are totally minimally invasive (RAMIE and MIE), we will focus on oncologic results in between the two techniques.

The difference between RAMIE and MIE might also be found in the postoperative fatigue of the surgeon. With RAMIE, the surgeon sits behind the console in an ergonomic better position than with MIE where the surgeon stands at the table. With reduced postoperative fatigue after RAMIE, the surgeon might be able to perform at a higher level during the rest of the week.

Aforementioned studies show the need for randomized controlled trials comparing RAMIE to conventional MIE. Such randomized controlled trials can only be performed in high volume centers for esophageal surgery, in which both RAMIE and MIE are performed on a regular basis with standardized protocols. Furthermore, surgeons in these trial hospitals should have passed the learning curve both for MIE and RAMIE [36]. Therefore only surgeons with enough experience can contribute to this trial. The minimum required experience is a minimum of 50 RAMIE to include patients in the RAMIE arm and a minimum of 50 MIE to include patients in the MIE arm.

Currently, there are two Asian multicenter randomized controlled trials recruiting esophageal squamous cell carcinoma patients comparing RAMIE to conventional MIE: the REVATE trial (ClinicalTrials.gov Identifier: NCT03713749) [37] and the RAMIE Trial (ClinicalTrials.gov Identifier: NCT04306458) [38]. Results for these trials are awaited with great interest and might answer the question whether RAMIE is superior to MIE for patients with esophageal squamous cell carcinoma in the Asian population. Together with the results of the ROBOT-2 trial, aforementioned trials might elucidate the role of RAMIE compared to MIE in the treatment of esophageal adenocarcinoma or adenocarcinoma of the gastroesophageal junction.

This is the first randomized controlled trial designed to compare robot-assisted minimally invasive thoraco-laparoscopic esophagectomy with conventional minimally invasive esophagectomy as surgical treatment for resectable esophageal adenocarcinoma in the Western World.

If our hypothesis is proven correct, robot-assisted minimally invasive thoraco-laparoscopic esophagectomy will result in improved lymphadenectomy compared to the conventional minimally invasive esophagectomy.

## Data Availability

The datasets analyzed during the current study are available from the corresponding author on reasonable request. The authors of the protocol will have access to the finale dataset and the publication of the data. The datasets will be presented in the main manuscript or additional supporting files, in machine-readable format (such as spreadsheets) whenever possible.

## Abbreviations

AE: Adverse events
ARDS: Acute Respiratory Distress Syndrome
CCI: Comprehensive Complication Index
CI: Confidence Intervals
DSMB: Data Safety Monitoring Board
ECCG: Esophageal Complication Consensus Group
ECOG: European Clinical Oncology Group
EORTC: European Organization for Research and Treatment
HE: Hybrid Esophagectomy
ICU: Intensive Care Unit
ID: Identification Number
IHM: In-hospital Mortality
ITT: Intention to Treat
MCDC: Modified Clavien-Dindo Classification
MCU: Medium Care Unit
MIE: Minimally Invasive Esophagectomy
OES: EORTC Criteria for esophageal Cancer
OP: Operation
OS: Overall Survival
OTE: Open Transthoracic Esophagectomy
PI: Principal Investigator
PVT: Psychomotor Vigilance Tests
QLQ-c30: Quality of Life questionnaire – core 30
QoL: Quality of Life
RAMIE: Robot Assisted Minimally Invasive Esophagectomy
SAE: Severe Adverse Events
SI: Secondary Investigator
UGIRA: The Upper-GI International Robotic Association
UICC: The Union for International Cancer Control
VAS: Visual Analog Scale
